# On the formation of inclusion complexes at the solid/liquid interface of anchored temperature-responsive PNIPAAM diblock copolymers with γ-cyclodextrin

**DOI:** 10.1007/s00396-017-4052-6

**Published:** 2017-03-22

**Authors:** Giuseppe Lazzara, Richard A. Campbell, Solmaz Bayati, Kaizheng Zhu, Bo Nyström, Tommy Nylander, Karin Schillén

**Affiliations:** 1 Division of Physical Chemistry, Department of Chemistry, Lund University, P.O. Box 124, SE-221 00 Lund, Sweden; 2 Department of Physics and Chemistry, University of Palermo, Viale delle Scienze, 90128 Palermo, IT Italy; 3 Institut Laue-Langevin, 71 avenue des Martyrs, 38042 Grenoble, France; 4 Akzo Nobel Surface Chemistry AB, Stenunge Allé 3, SE-444 85 Stenungsund, Sweden; 5 Department of Chemistry, University of Oslo, P.O. Box 1033, Blindern, 0315 Oslo, Norway

**Keywords:** Inclusion complex, Cyclodextrin, Poly(*N*-isopropylacrylamide), Thermoresponsive block copolymer, Solid/liquid interface, Reflectometry

## Abstract

The thermal responsive behavior of adsorbed layers of diblock copolymers of poly(*N*-isopropylacrylamide) (PNIPAAM) and poly((3-acrylamidopropyl)trimethylammonium chloride) (PAMPTMA(+)) with γ-cyclodextrin (γ-CD) at the solid/liquid interface has been investigated using three in situ techniques: null ellipsometry, quartz–crystal microbalance with dissipation monitoring, and neutron reflectometry. The measurements provided information about the adsorbed amounts, the layer thickness, hydration and viscoelastic properties, and the interfacial structure and composition. The copolymers adsorb to silica with the cationic PAMPTMA(+) blocks sitting as anchors in a flat conformation and the PNIPAAM chains extending into the solution. The copolymer system alone exhibits reversible collapse above the lower critical solution temperature of PNIPAAM. The addition of γ-CD to pre-adsorbed copolymer layers results in a highly extended conformation as well as some loss of copolymer from the surface, which we discuss in terms of the formation of surface-invoked lateral steric repulsion of formed inclusion complexes.

## Introduction

Polyrotaxanes (or inclusion complexes) are supramolecular assemblies formed by threading a polymer chain through several ring-shaped molecules like cyclodextrin (CD) [1]. Pseudopolyrotaxane is a type of inclusion complex that lacks stoppers at the end of the polymer chains. Cyclodextrins are cyclic oligosaccharides formed by glucopyranose units [2]. They have a truncated cone shape with a hollow, hydrophobic cavity, which may incorporate more or less hydrophobic solutes. They also have negligible toxicity, high chemical stability, and water solubility. CDs typically contain a number of glucose monomers ranging from six to eight units, which are denoted with the prefix α, β, or γ, respectively. Pseudopolyrotaxanes and polyrotaxanes formed by block copolymers and CD molecules are extensively investigated because they can form self-assembly supramolecular structures that can be tuned by using external stimuli, like temperature and pH [3–6]. Controlling the structure requires understanding of the mechanisms, which although challenging, opens up the possibilities for designing molecular switches or smart nanomaterials to be used in, e.g., bioanalysis, bioseparation, and in drug and gene delivery systems [1, 4, 7, 8]. The CD cavity size generally controls the block selectivity while using chemically modified CDs, such as alkylated CD, one can alter the CD/macromolecule stoichiometry. Even if the bulk studies on block–copolymer/CD supramolecular assemblies, including host–guest supramolecular polymers and CD-containing polymers, are numerous in literature, see for instance refs [5, 7, 9–16], there is only a minor knowledge concerning their behavior at the solid–liquid interface [17]. The need for so-called responsive surfaces where the properties, including release of molecules from the surface layer, are triggered by environmental changes, like temperature, has increased with fast development in the nanomaterials field.

Previously, we have investigated interactions in aqueous mixtures of γ-cyclodextrin (γ-CD) and a thermosensitive cationic diblock copolymer composed of poly(*N*-isopropylacrylamide) (PNIPAAM) and poly((3-acrylamidopropyl)trimethylammonium chloride) (PAMPTMA(+)) denoted by PNIPAAM_24_-*b*-PAMPTMA(+)_9_ [18]. It was found that γ-CD–copolymer inclusion complexes form both in solution and in the solid state as a precipitate, depending on the γ-CD concentration, at ambient temperature. A precipitate is evidence of the formation of crystalline pseudopolyrotaxanes [10]. The ^1^H-NMR signals detected for copolymer-CD mixtures in D_2_O revealed that the bulky cationic PAMPTMA(+) block does not interact with the γ-CD while the chemical shift of the protons in the PNIPAAM block were shifted towards lower fields. This implies that only the PNIPAAM block is enclosed into the CD cavity, and the stoichiometry was found to be close to two NIPAAM units per γ-CD molecule. This in turn reflects the close packing of the threaded γ-CD molecules along the polymer chain. The structure of the solid inclusion complexes obtained by synchrotron radiation powder X-ray diffraction experiments was consistent with a columnar structure with compact arrangement of the γ-CD molecules in head-to-head/tail-to-tail fashion in accordance with the NMR results. A columnar structure has previously been found for pseudopolyrotaxanes composed of γ-CD and poly(ethylene glycol) [19]. PNIPAAM is a thermoresponsive polymer with a sharply reduced solubility in water upon heating at a well-defined temperature, the so-called lower critical solution temperature (LCST), due increased hydrophobicity and weakening of the water–amide hydrogen bonds [20–22]. This general phenomenon of changing the apparent quality of the solvent is manifested in a coil-to-globule collapse of individual polymer chains, releasing bound water molecules [23, 24]. Such a transformation is followed by an intermolecular aggregation, and eventually, the polymer phase separates to form particles. For solutions of PNIPAAM in pure water, the coil-to-globule transition is exhibited around 32 °C, which depends on concentration and, in case of short polymers, depends on the molecular weight, polydispersity, end effects, branching, and tacticity [22, 25].

Upon increasing temperature, the diblock copolymer system investigated in our solution study also showed a clouding behavior, and we observed a dethreading of the γ-CD molecules from the PNIPAAM block by using steady-state fluorescence spectroscopy [18]. In light of the interesting solution properties of the PNIPAAM_24_-*b*-PAMPTMA(+)_9_–γ-CD copolymer system, we have here determined whether the corresponding interactions were present for the same system at a solid/liquid interface (a flat silica surface). The overall aim is to understand and produce functional surfaces that respond on the molecular level to external stimuli. For this purpose, we have revealed the detailed structural change of such copolymer layers upon addition of γ-CD and how the formation of confined inclusion complexes at the solid/liquid interface affects the switchable thermal response. The chosen surface-sensitive, in situ methods of investigation are null ellipsometry, quartz crystal microbalance with dissipation monitoring (QCM-D), and neutron reflectometry (NR), which together are capable of providing quantitative information on the surface excess, layer thickness, hydration and elasticity, and interfacial structure. Previously, other groups have successfully used these techniques to study the heat-induced dehydration and collapse of PNIPAAM brushes at planar surfaces, see, e.g., refs [26–28] and the refs therein.

## Materials and methods

### Materials

**Chart 1:**
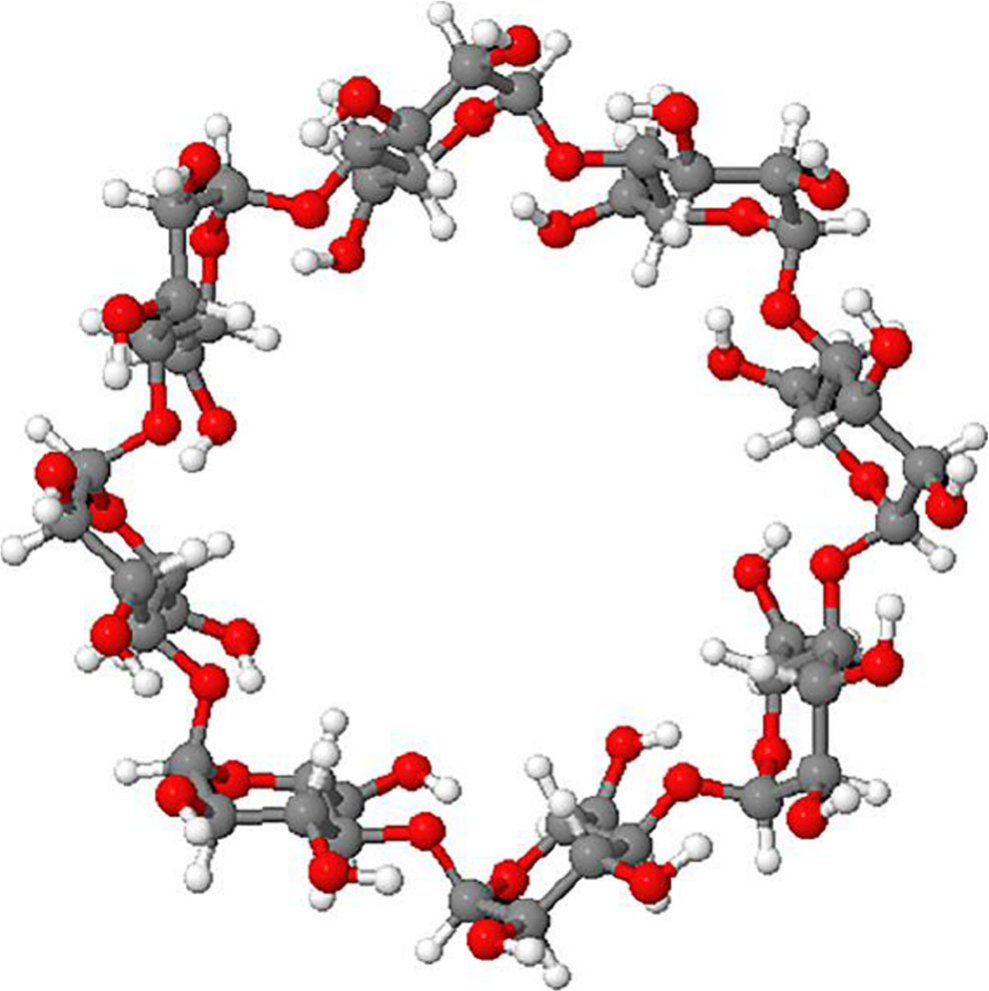
Molecular structure of γ-cyclodextrin. Top view from the larger opening. Color codes: carbon (*gray*), oxygen (*red*), and hydrogen (*white*)

γ-Cyclodextrin with a molar mass of 1296 g mol^−1^ was purchased from Sigma-Aldrich (Germany) and was used as received. The chemical structure of γ-CD is given in Chart 1.

Thermogravimetric analysis was used to determine the water content of the γ-CD sample as 15.3 wt%. Solutions were prepared using water purified by a Milli-Q system (Millipore Corporation, Bedford, MA), D_2_O, or a mixture of the two, depending on the experimental technique. D_2_O (99.8% isotopic purity) was acquired from Armar Chemicals (Döttingen, Switzerland) or Eur-isotop (Saclay, France). Stock solutions of copolymer or γ-CD were left to equilibrate overnight. The pre-mixed solutions were equilibrated for a week prior to use. All solutions used in the different experiments contained 1 mM of sodium chloride, NaCl (Merck, at least 99% purity, PA). It should be noted that in solution, inclusion complexes coexist with self-assembled aggregates of neat γ-CD as observed from previous dynamic light scattering studies [18]. Furthermore, these aggregates are few in comparison with the complexes after at least 24 h of equilibration of the mixed solution. These aggregates may have an affinity to the silica surface similar to those of α-CD [29]; however, because of the long equilibrium time of the mixtures used in this study, the remaining neat γ-CD aggregates are assumed to be too few to affect the experimental results.

### Synthesis of poly(*N*-isopropylacrylamide)_*n*_-*b*- poly((3-acrylamidopropyl)trimethylammonium chloride)_*m*_

**Chart 2:**
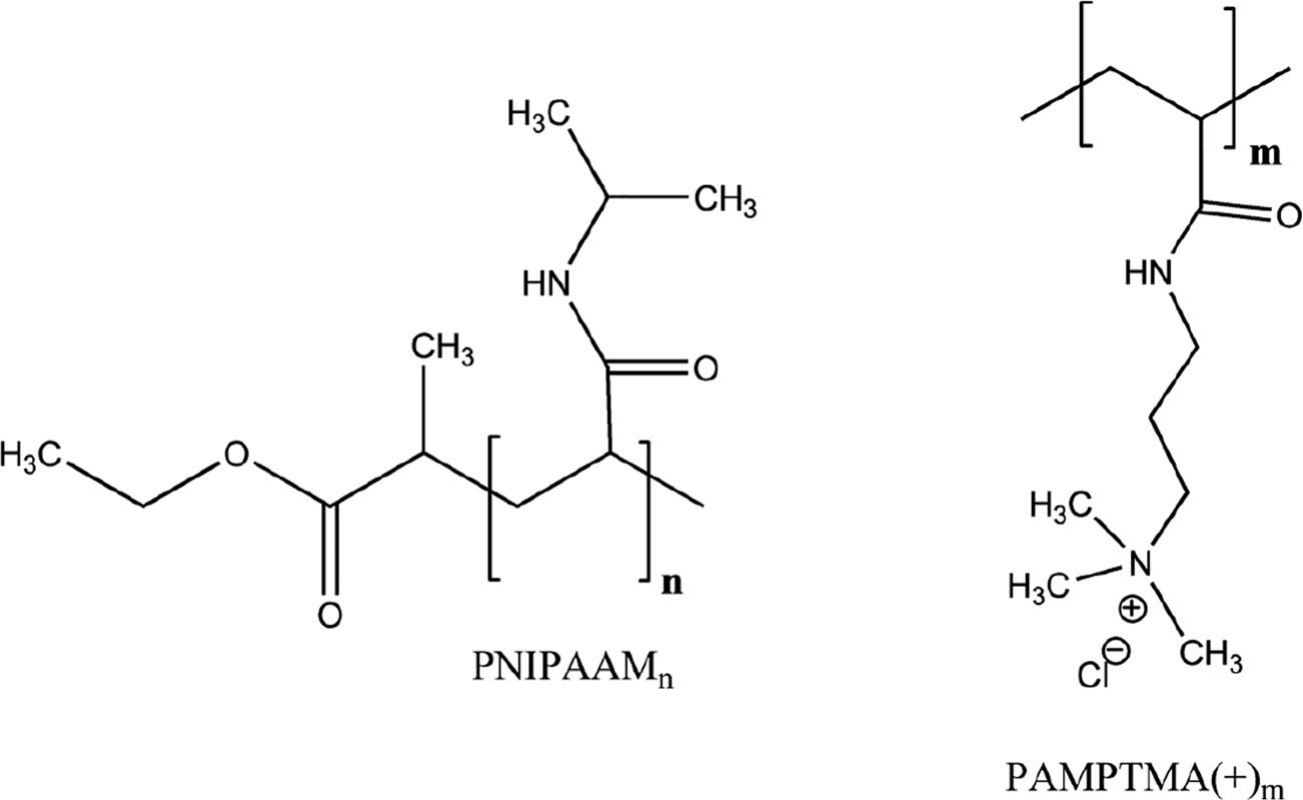
Molecular structure of the nonionic and the cationic blocks of the diblock copolymer in this study (see refs [18, 31] for block length characterization): poly(*N*-isopropylacrylamide) (PNIPAAM_*n*=24 or 41_) and poly((3-acrylamidopropyl)trimethylammonium chloride PAMPTMA(+)_*m*=9 or 24_)

The cationic diblock copolymers were synthesized by means of atom transfer radical polymerization (ATRP) via a simple “one-pot,” two-step procedure and purified using the methodology described in detail in ref. [30]. The composition of the two copolymers are PNIPAAM_24_-*b*-PAMPTMA(+)_9_ or PNIPAAM_41_-*b*-PAMPTMA(+)_24_ as previously determined from their corresponding ^1^H-NMR spectra [18, 31]. The nominal molar mass *M*_*n*_(NMR) is 5477 or 10,227 g mol^−1^, respectively. The molecular structures of the individual blocks are displayed in Chart 2.

The PNIPAAM block of the PNIPAAM_24_-*b*-PAMPTMA(+)_9_ copolymer is expected to have a contour length (*R*_max_) of 60.4 Å, i.e., the maximum end-to-end distance of the chain to the all-*trans* conformation. It is calculated from *R*_max_ = 24 ∙ *n*_*C* − *C*_ ∙ *l* ∙  sin (*θ*/2), where *n*_*C* − *C*_, *l*, and *θ* are the number of C–C bonds in the repeating unit, C–C bond length (*l* = 1.54 Å), and C–C bond angle (109.5°), respectively. Similarly, *R*_max_ of the PAMPTMA(+) block is 22.7 Å. For the NR experiments, a diblock copolymer with a slightly different composition PNIPAAM_41_-*b*-PAMPTMA(+)_24_ with a PNIPAAM block with *R*_max_ = 103.1 Å and a cationic block length of 60.4 Å was used (*M*_*n*_(NMR) = 10,227 g mol^−1^). The LCST has been determined by differential scanning calorimetry to be 45.6 and 43.1 °C for PNIPAAM_24_-*b*-PAMPTMA(+)_9_ and PNIPAAM_41_-*b*-PAMPTMA(+)_24_, respectively, whereas LCST of the homopolymers, PNIPAAM_30_ and PNIPAAM_47_, is 38.6 and 37.9 °C, respectively [18, 31]. The reason for the lower LCST for the latter copolymer is mainly due to the significantly longer PNIPAAM block. In our work, the PAMPTMA(+) block is expected to be attached to the surface and, therefore, it is likely that it does not influence the LCST of the PNIPAAM chain. For this reason, we think that the results recorded with the different methods are qualitatively comparable.

### Null ellipsometry

The adsorbed amount and thickness of adsorbed layers of both the PNIPAAM_24_-*b*-PAMPTMA(+)_9_ copolymer alone and mixtures of PNIPAAM_24_-*b*-PAMPTMA(+)_9_ with γ-CD onto silica surfaces were measured in situ using an automated Rudolph research thin-film null ellipsometer, type 43603-200E. A xenon arc lamp with a filter for a wavelength of 4015 Å was employed as a light source in these measurements. A 5-mL thermostated cuvette equipped with a magnetic stirrer at about 300 rpm was used as the sample cell. The cell allowed the temperatures to be set at 20 and 40 °C, which is well below and above the LCST of PNIPAAM with a length similar to the PNIPAAM block of the copolymer.

The recorded values of the ellipsometric angles, Ψ and ∆, correspond to the relative change in amplitude and phase shift upon reflection of polarized light at an interface, respectively. They are related to the complex reflectivity coefficients for light components polarized parallel and perpendicular to the plane of incidence, *r*_*p*_ and *r*_*s*_, respectively

1 $$ \frac{r_p}{r_s}= \tan \Psi {e}^{i\varDelta } $$

The silicon wafers ([100] crystal plane, *p*-type, boron doped, and resistivity of 1–20 Ω cm) were obtained from Department of Chemistry, IFM, Linköping University, Sweden. The silicon was thermally oxidized under oxygen at 920 °C for 1 h, followed by annealing and subsequent cooling under argon flow. The silicon dioxide, SiO_2_, layer achieved by this method is about 300 Å. These oxidized wafers were cut into slides with a width of 12 mm. Before use, they were cleaned according to the protocol described in ref. [32] and thereafter stored in ethanol. Prior to the start of the experiments, the wafers were dried under vacuum and then treated in an air plasma cleaner (Harrick Scientific Corp., model PDC-3XG) for 5 min.

The measurements were performed based on the protocol described by Tiberg and Landgren [32]. The ellipsometric angles of the bare substrate were first measured in air and aqueous solution. These allowed us to calculate the complex refractive index of silicon (*N*_Si_), the real refractive index (${n}_{\mathrm{Si}{\mathrm{O}}_2\kern-0.4em }$), and the thickness (${d}_{\mathrm{Si}{\mathrm{O}}_2\kern-0.4em }$) of the oxide layer in each case by applying a three-stratified optical model [33].

The adsorption experiments were performed by flowing a NaCl solution of 1 mM through the sample cell at a continuous flow rate of 3.6 mL min^−1^ and thermostated at 20 °C in the beginning of the experiment. After reaching a constant baseline, a copolymer solution containing 1 mM NaCl was added by pipetting an appropriate volume of the stock solution (250 μL) to a final concentration of 0.005 wt% and Ψ and Δ were recorded as a function of time until no further change was detected. Thereafter, the cell was rinsed with a 1 mM NaCl solution by means of a peristaltic pump (Ole Dich Instrumentmakers ApS, Hvidovre, Denmark) at a rate of 1.5 mL min^−1^. Next, a stock solution of cyclodextrin was injected to a final concentration of 0.05 wt%, followed by measurements of Ψ and Δ and another rinsing step. The temperature was set in the range 20–40 °C.

The ellipsometry data evaluation was carried out using a program based on McCrackin’s approach assuming a planar interface with one adsorption layer, i.e., a four-layer model in total involving silicon, SiO_2_, adsorbed material, and an isotropic solvent [33]. The adsorbed amount, Γ, was obtained from the calculated values of the mean refractive index, *n*_*f*_, and the thickness, *d*_*f*_, [34] using de Feijter’s expression

2 $$ \Gamma =\frac{\left({n}_f-{n}_0\right){d}_f}{\mathrm{dn}/\mathrm{dc}} $$

where *n*_0_ is the refractive index of the bulk solution (in this case it is very close to water value as the experiments were performed under dilute solution conditions) and dn/dc is the refractive index increment of the adsorbed material.

### Quartz crystal microbalance with dissipation monitoring

In QCM-D, the changes in mechanical resonance of piezoelectric single-crystalline quartz subject to an external electric field are measured. The QCM-D technique is very sensitive to changes in mass adsorbed/deposited on the surface. The quantity obtained is the wet mass, i.e., the adsorbed material including solvent in a surface layer. The energy dissipation factor is another important output from the QCM-D measurements. This is a measure of the viscoelastic properties of the layer and is determined by measuring the decay in response, when a pulse at the resonance frequency is applied to the crystal. As the resonance frequency is very sensitive to the temperature, the temperature response was measured also for the bare crystal before the adsorbing components were added.

QCM-D measurements were performed with a Q-SENSE E4 system, equipped with four liquid sample cells that allow four experiments to be performed in parallel. A detailed description of the method can be found by Rodahl et al. [35]. Measurements were performed with a continuous flow of 50 μL min^−1^ of the solution through the sample cell by using a peristaltic pump (Ismatec, Zürich, Switzerland). Two types of experiments were performed, first by pre-adsorption of PNIPAAM_24_-*b*-PAMPTMA(+)_9_ from a solution of 0.05 wt% followed by addition of a γ-cyclodextrin solution of 0.1 wt% and secondly by adsorption of inclusion complexes from a premixed solution of γ-CD (5 wt%) and PNIPAAM_24_-*b*-PAMPTMA(+)_9_ (0.5 wt%). The measurements were carried at 20 and 40 °C, respectively, and the data were corrected for the signal of the bare surface to compensate for the temperature effects on the crystal acoustic properties.

The sensor crystals, coated with SiO_2_ and with a resonance frequency of 5 MHz (QSX-303), were purchased from Q-Sense (Gothenburg, Sweden). The cleaning procedure involved treating them in a plasma cleaner for 10 min and then to soak them in 1 wt% SDS solution for at least 1 h. Afterwards, they were thoroughly rinsed with Milli-Q water and were then stored in ethanol. Prior to the experiments, the surfaces were dried under vacuum and were subsequently treated in the plasma cleaner for 5 min.

The wet mass, ∆*m*, of the copolymer and the γ-CD–copolymer inclusion complexes adsorbed to silica was calculated by applying the Sauerbrey expression

3 $$ \varDelta m=\frac{C}{o_n}\varDelta f $$

where *C* ≈ 17.7 ng Hz^−1^ cm^−2^ for a 5 MHz crystal and *o*_*n*_ is the overtone number.

This expression is a good approximation for rigid, thin, and evenly distributed adsorbed films as discussed by Höök et al. [36]. It has been shown that as long as the damping is low (elastic layers), relative differences can still be trusted with high accuracy [37] and that this was the case that was confirmed by comparing Δ*f* and Δ*D* for the different harmonics. In the present study, the addition of copolymer and γ-CD only cause minor changes in the dissipation (Δ*D* ≈ 1) and the fact that we compare mainly relative changes, we use Eq. 3 to quantify the adsorbed layer. It is important to point out that even an acoustically rigid film can contain substantial amount of solvent that is included in the mass.

### Neutron reflectometry

The theory and applicability of NR have been described in the review by Thomas [38]. The reflectivity profile is the ratio of intensity of the specular reflection of a neutron beam (corrected for background scattering) to that of the incident beam with respect to momentum transfer defined as *Q* = (4*π* sin *θ*)/*λ*, where *θ* is the grazing incidence angle on the sample and *λ* is the wavelength. To cover the appropriate *Q* range, two angles (0.62° and 3.78°) were employed using the horizontal reflectometer at the Institut Laue-Langevin (Grenoble, France) [39]. The time-of-flight instrument was operated with a resolution in *Q* of 6% and a wavelength range of 4–24 Å in the reduced data. This allowed for collection of the data in the *Q* range 0.006 to 0.2 Å^−1^. The substrates used were (111) silicon crystals (Siltronix, France) with the dimension 5 × 5 × 1 cm. They were cleaned according to the protocol described in ref. [40]. After cleaning the crystals, they were placed in a liquid flow cell made of Teflon with a volume of about 5 mL. The sample cells are described in detail elsewhere (see ref. [40]) and allowed for accurate temperature control (±0.1 °C) by means of an external thermostat bath. The sample additions were made by flowing through at least 20 mL (i.e., four times the cell volume) to ensure sufficient exchange of the bulk solution. Two temperatures were used, 20 and 46 °C.

The experiments were carried out by adsorbing first copolymer and then γ-CD to the silica surface. A longer copolymer was used for NR than for ellipsometry and QCM-D: it had 24 AMPTMA(+) units and 41 NIPAAM units per molecule, and this choice was made as a result of the reasons described in the “Results and discussion” section below. Three contrasts were investigated: H_2_O, D_2_O, and a mixture of the two. The concentrations of copolymer and γ-CD were 0.08 and 1.0 mg mL^−1^ (or 0.1 and 1.0 wt%), respectively. A background salt concentration of 1 mM NaCl was used.

Neutron scattering exploits the fact that different isotopes scatter neutrons differently. Most notable is the large difference between deuterium and hydrogen, which means that D_2_O and H_2_O have very different scattering length densities. The scattering length density, *ρ*, of a material can be expressed as

4 $$ \rho =\frac{\sum {n}_i{b}_i}{V_{\mathrm{m}}} $$

where *n* is the number of a given nucleus *i*, *b* is its coherent scattering cross section and *V*_m_ is the molecular volume [41].

Table 1 shows the scattering length densities for the substrate, solvents, and chemical compounds used in this study. Some experimental problems with the solvent pump were experienced during the experiment unfortunately, and the fitted scattering length density of the mixed D_2_O/H_2_O solvent had to be varied in the range 1.45–1.76 × 10^−6^ Å^−2^. Note that the scattering length density of CD molecules depends on the isotopic contrast of the solvent because the hydrogen atoms in the hydroxyl groups are labile [29]. Exchange of 24 atoms per molecule was taken into consideration together with a used molecular volume of 1352 Å ^3^ [42]. The fitted interlayer roughness between the silicon and the SiO_2_ was 3 Å, and the residual background used in the fits were 10^−6^ for the data recorded in D_2_O and 5 × 10^−7^ for the data recorded in the other solvent contrasts.

**Table 1:** Scattering length densities (*ρ*) of the materials used in the evaluation of the neutron reflectivity data

Material	*ρ* × 10^6^/Å^2^
PNIPAAM	0.761
PAMPTMA(+)	0.508
γ-CD in D_2_O	3.73
γ-CD in mixed D_2_O/H_2_O	2.57
γ-CD in H_2_O	1.86
Si	2.07
SiO_2_	3.47
H_2_O	–0.56
D_2_O (fitted)	6.34

PNIPAAM = poly(*N*-isopropylacrylamide), PAMPTMA(+) = poly((3-acrylamidopropyl)trimethylammonium chloride), γ-CD = γ-cyclodextrin

The neutron reflectivity profiles of the block copolymer sample at 20 and 46 °C were analyzed by fitting simulated reflectivity profiles to the experimental data in Motofit [43], which uses the Abeles optical matrix method [44] to calculate the reflectivity of thin layers and enables global fitting of data sets of different isotopic compositions. The model required to fit the data had two adsorption layers, i.e., a five-layer model in total involving silicon, SiO_2_, the cationic PAMPTMA(+) anchor block, the PNIPAAM extended block in aqueous bulk solution, and solvent. The stoichiometry of the two blocks was then preserved with respect to their relative volumes to within a maximum error of 20%, thus allowing a small contribution for mixing of a given block into the adjacent layer. Note that it was not possible to fit, the experimental data using a model with a single adsorbed layer.

The fitting parameters used for each layer were *ρ*, the thickness, *d*, the interfacial roughness, *δ*, and the solvent volume fraction, *ϕ*. The adsorbed amount of a given component can be calculated from the fits using

5 $$ \Gamma =\frac{\rho d\left(1-\phi \right){M}_{\mathrm{w}}}{\sum {n}_i{b}_i\cdot {N}_{\mathrm{av}}} $$

where *M*_w_ is its molecular weight, and *N*_av_ is the Avogadro constant.

## Results and discussion

### Adsorbed amount and layer thickness (null ellipsometry)

Null ellipsometry was first employed to monitor the thermal responsive properties of PNIPAAM_24_-*b*-PAMPTMA(+)_9_ copolymer in 1 mM NaCl, in the absence and presence of γ-CD and in situ at the silica/solution interface. We examined several facets of the system to reveal the structural response: adsorption of polymer at 20 °C, heating to 40 °C, cooling back to 20 °C, exposure of γ-CD to the polymer layer, and then further heating and cooling cycles.

**Fig. 1:**
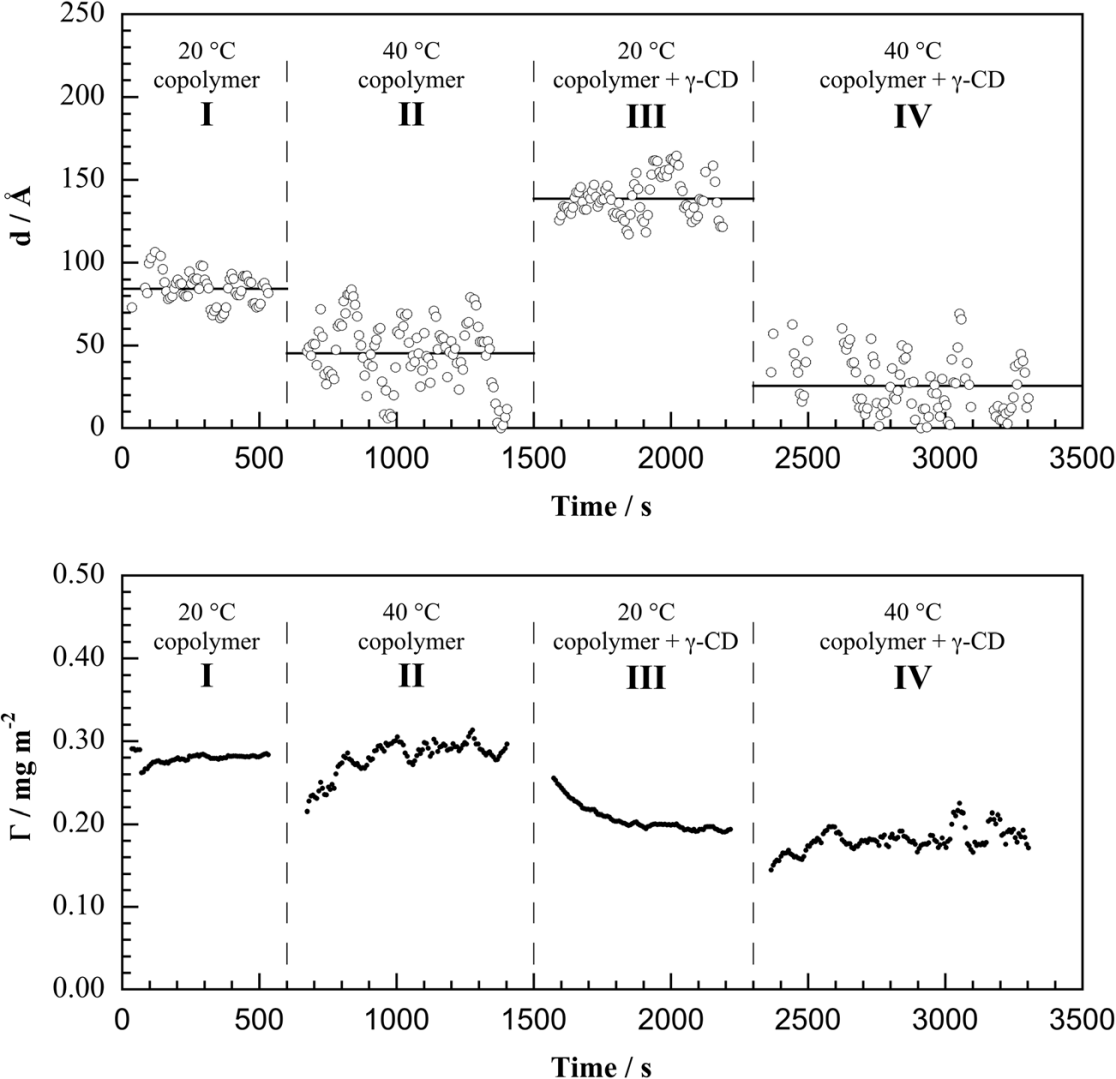
Layer thickness *d* (*upper panels*) and adsorbed amount Γ (*lower panels*) determined by null ellipsometry, as a function of time after addition of PNIPAAM_24_-*b*-PAMPTMA(+)_9_ copolymer to silica at 20 °C (region I), followed by a temperature increase to 40 °C (region II), followed by addition of γ-CD at 20 °C (region III), and then followed by a temperature increase to 40 °C (region IV). The macromolecular concentrations are as follows: 0.005 wt% PNIPAAM_24_-*b*-PAMPTMA(+)_9_ in 1 mM NaCl and 0.05 wt% γ-CD in 1 mM NaCl

Representative data as a function of time after exposing 0.005 wt% PNIPAAM_24_-*b*-PAMPTMA(+)_9_ in 1 mM NaCl to the silica surface at 20 °C are shown in region I of Fig. 1, where the layer thickness *d* (upper panel) and adsorbed amount Γ (lower panel).

The values of *d* and Γ reach a plateau (or mean) value of 84 ± 9 Å and 0.28 ± 0.01 mg m^−2^, respectively, within minutes (errors given as ±standard deviation, std). These values correspond to a volume fraction of copolymer in the adsorbed layer of about 0.029, if we assume a partial specific volume of the copolymer of 0.87 cm^3^ g^−1^ (the value for PNIPAAM at 20 °C) [24]. The driving force for the adsorption process is the electrostatic interaction between the positively charged PAMPTMA(+) blocks and the negatively charged silica surface, where the charged blocks compensates the surface charge. As such, the interfacial structure may be expected to be a relatively dense layer of the anchor block with a less dense layer of the extended block attached. Here, it should be noted that also PNIPAAM has a slight affinity to silica; thus, it cannot be ruled out that some of the PNIPAAM blocks are also attached to the silica. Null ellipsometry does not have the depth of information to elaborate on the surface structure, but we will return to this issue later with the analysis of the NR data. Even so, the modest adsorbed amount in these measurements indicates a low segment density on the surface and a highly hydrated layer. In this case, the determination of *d* is close to the detection limit, which explains the high scatter in the data.

A calculation of the surface density, above which the block copolymer chains are overlapping in the adsorbed layer, can be estimated from ${\sigma}_{ol}=1/\left(\pi {R}_{\mathrm{F}}^2\right)$, where *R*_F_ is the Flory radius for a PNIPAAM polymer chain in a good solvent. The Flory radius of the PNIPAAM block is calculated from *R*_F_ = *l*_eff_ *n* ^0.6^, where *n* is the number of repeating units and *l*_eff_ is the effective unit length (*l*_eff_ = *n*_*C* − *C*_ · *l* = 0.308 nm) [45]. Thus, for a PNIPAAM_24_ homopolymer in water at good solvent conditions below LCST, *R*_*F*_ is 2.07 nm, which gives an overlap surface density of 7.40 × 10^16^ m^−2^. The measured mean surface density, *σ*, at 20 °C may be calculated from *σ* = Γ*N*_*A*_/*M*_*n*,PNIPAAM_, where Γ in this case is equal to 0.28 mg m^−2^ (Table 1), and *M*_*n*,PNIPAAM_ is the molar mass from NMR of the PNIPAAM_24_ block (i.e., 2716 g mol^−1^). A value smaller than the overlap surface density is obtained (*σ*/*σ*_*ol*_ = 0.84). This result implies that the average distance between the diblock copolymers adsorbed on the surface is larger than the extension of the same polymer in solution, and thus the adsorbed polymers are moderately stretched, as *σ*/*σ*_*ol*_ > 1 is valid when the polymer chains are forced to be fully stretched into such structure. Another way to estimate the polymer conformation on the surface is to compare the chain extension (2*R*_F_) with the distance between adsorbed polymer chains at the surface, *s*, calculated from the 20 °C data (*s* = 4.53 nm). When *s* > 2*R*_F_, the polymers are in the nonoverlapping regime (the “mushroom” regime) and whereas when *s* ≪ 2*R*_F_, the polymers are in the regime where they overlap (the “brush” regime) [45]. Also, this calculation indicates that the polymers in the layer are not fully stretched. This may be explained that, in the present system, it is likely that the anchoring PAMPTMA(+) block is adsorbed in a flat configuration and thus the limiting factor of the adsorbed amount is the lateral repulsion between the adsorbed species as commonly observed for cationic polymers. Furthermore, our calculation above indicates that, similar to PNIPAAM homopolymers [27, 46, 47], there exists an affinity of the PNIPAAM block to the silica surface also for this block copolymer system. The PNIPAAM chains are adsorbed with a conformation that depends on the amount of space left at the surface as described in ref. [48]. After the measurements at 20 °C, the cell was rinsed with 1 mM NaCl solution. This process resulted in no measurable desorption of copolymer, which demonstrates the irreversibility of the adsorption process.

As expected, a temperature increase to 40 °C above the LCST of the PNIPAAM block resulted in a significant decrease in mean layer thickness (46 ± 20 Å; Fig. 1, region II). Again, there was no significant loss of material from the surface associated with the collapse of the layer, which shows that there is no sterically induced detachment; the initial increase in adsorbed amount is an experimental artifact due to the change of temperature. The temperature was then lowered to 20 °C to examine the reversibility of the process. We found that the layer thickness increased to the same value as before heating to 40 °C. This validation of the reversibility of polymer conformation changes allows us to examine the switching process in the presence of γ-CD.

After equilibration at 20 °C, 0.05 wt% γ-CD was exposed to the adsorbed polymer layer, which caused extensive swelling to a mean layer thickness of 139 ± 12 Å (Fig. 1, region III). We may infer that the increase in layer thickness is primarily due to formation of inclusion complexes, i.e., threading of the PNIPAAM chains through the cyclodextrin molecules to form the arrangement of 2 NIPAAM units/γ-CD molecule previously observed in solution and in the solid state [18]. The mean layer thickness increases significantly as a result of the interaction, which is consistent with the specific interaction of γ-CD with the anchored polymer molecules, and may indicate that the anchor blocks bound to the silica adopt a less flat conformation. In fact, the value of the mean layer thickness (139 Å) now exceeds that of the entire polymer molecule (83 Å), which is physically unrealistic and probably exposes the limited accuracy of the thickness information extracted from ellipsometry when the layer structure is more intricate than a uniform layer and contains multiple components. Nevertheless, comparisons of the relative mean layer thicknesses are useful, and the accuracy in the measured adsorbed amounts is greater. As the adsorbed amount of material is less in the presence (steady-state value of 0.19 mg m^−2^) than the absence (steady-state value of 0.28 mg m^−2^) of γ-CD at 20 °C, it is clear that the addition of γ-CD solution resulted in removal of some of the copolymer from the surface. Unfortunately, ellipsometry does not have the resolution in composition to determine how much polymer was removed or how much γ-CD was incorporated into the layer. Even so, this result clearly indicates that the effect of the formation of inclusion complexes makes it less favorable for the copolymer to stay attached at the solid/liquid interface. Possible reasons for the destabilization of the polymer are surface-invoked lateral steric repulsion of the inclusion complexes or competitive adsorption of γ-CD to the silica.

The temperature was thereafter increased to 40 °C (Fig. 1, region IV). The mean layer thickness decreased to 26 ± 17 Å, while the adsorbed amount remained essentially the same (0.20 ± 0.2 mg m^−2^ compared to 0.18 ± 0.1 mg m^−2^; Table 2) albeit possibly with a small loss of material from the surface; note the small initial increase due to the temperature equilibration effect as mentioned above. This possible small loss of material is qualitatively consistent with the dethreading of CD molecules from PNIPAAM chains upon heating them to above the LCST, which we have previously demonstrated in the bulk solution [15], but in the present data, the effect, if real, is within the stated experimental uncertainties. The mean layer thickness decreased as compared to the thickness measured at 40 °C before γ-CD was added, which demonstrates that the underlying nature of the PNIPAAM blocks to undergo collapse upon an increase in temperature to above the LCST is preserved following the interaction of γ-CD molecules with the adsorbed copolymer layer. The data from the ellipsometry measurements show that γ-CD molecules interact with the polymer when adsorbed to silica, removing some of the polymer in the process, but the technique is not able to detect the location of CD. Furthermore, this technique can only detect loss of material and not discriminate between CD and polymer at the surface. Hence, additional techniques and experimental methodologies were applied to provide more information.

**Table 2:** Experimental data obtained from ellipsometry and QCM-D measurements performed by sequential addition of PNIPAAM_24_-*b*-PAMPTMA(+)_9_ copolymer and γ-cyclodextrin (γ-CD) to silica surfaces

	Copolymer 20 °C	Copolymer 40 °C	Copolymer + γ-CD 20 °C	Copolymer + γ-CD 40 °C
*d*/Å (ellipsometry)	84 ± 9	46 ± 20	139 ± 12	26 ± 17
Γ/mg m^−2^ (ellipsometry)	0.28 ± 0.01	0.28 ± 0.02	0.20 ± 0.02	0.18 ± 0.01
∆*f*_3_/Hz (QCM-D)	−22.9 ± 0.2	−19.8 ± 0.1	−21.3 ± 0.2	−16.7 ± 0.2
∆*D*_3_ × 10^−6^/(QCM-D)	1.30 ± 0.08	0.97 ± 0.02	≈0	≈0
∆*m*/mg m^−2^ (QCM-D)	4.00 ± 0.05	3.50 ± 0.05	3.70 ± 0.05	2.91 ± 0.05

*d* = layer thickness, Γ = adsorbed amount, ∆*f*_3_ = frequency shift of third overtone of the resonance frequency, ∆*D*_3_ = dissipation difference from the third overtone, ∆*m* = wet mass difference calculated from Eq. 3 using the third overtone

### Layer hydration and elasticity (QCM-D)

We performed QCM-D experiments on the PNIPAAM_24_-*b*-PAMPTMA(+)_9_—γ-CD system to examine (1) the changes in copolymer hydration and viscoelastic properties when responding to temperature changes and (2) whether the surface properties are dependent on the pathway with which the layer is created. To achieve the latter aim, the experiments were carried out via two different experimental pathways: (1) by pre-adsorbing the diblock copolymers to a silica surface and thereafter sequential addition of γ-CD (*sequential* adsorption, as performed above with ellipsometry) or (2) by using a pre-mixed solution consisting of γ-CD–copolymer inclusion complexes (*complex* adsorption).

#### Sequential adsorption

**Fig. 2:**
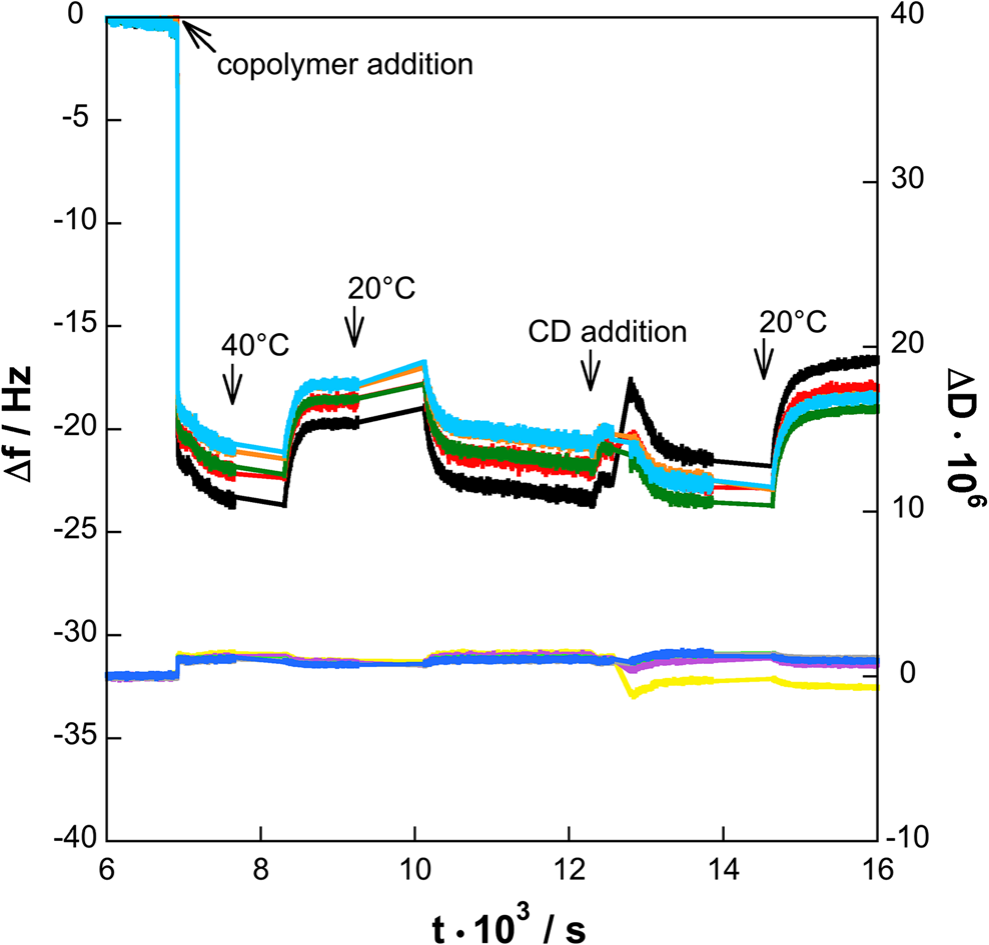
Changes in frequency shifts Δ*f*: from *bottom*, F3 (black), F5 (*red*), F7 (*green*), F11 (*orange*), and F13 (*light blue*) and dissipation differences Δ*D*: D3 (*yellow*), D5 (*magenta*), D7 (*light green*), D11 (*gray*), and D13 (*blue*), at different overtones (overtone numbers *o*_*n*_ = 3, 5, 7, 11, and 13) of the resonance frequency determined by QCM-D. The PNIPAAM_24_-*b*-PAMPTMA(+)_9_ (0.05 wt%) and the γ-CD (0.1 wt%) solutions containing 1 mM NaCl were added sequentially to the silica surface. The time of addition, change of temperature, and rinsing are indicated by *arrows*

Figure 2 presents the frequency shift ∆*f* and dissipation difference ∆*D* versus time, while changing the temperature (overtone numbers *o*_*n*_ = 3, 5, 7, 11, and 13) for the sequential adsorption approach.

Both ∆*f* and ∆*D* have been corrected relative to the temperature behavior of the bare surface; this process was only possible at constant temperature; hence, data during temperature changes are represented with lines. The adsorption of the copolymer (0.005 wt% with 1 mM NaCl) onto the silica surface results in a decrease in frequency and can be used to calculate the wet mass change, ∆*m* from Eq. 3. The data calculation from the 3rd overtone is given in Table 2.

The calculated values of the wet mass from the QCM-D measurements at 20 °C are about one order of magnitude larger than the values of the optical mass obtained from ellipsometry, 4.00 ± 0.05 and 0.28 ± 0.01 mg m^−2^, respectively. While considering the uncertainty in the mass calculations using Eq. 3 as discussed above, we can estimate the degree of hydration by combining the QCM-D and ellipsometry data and then obtain a volume fraction of 0.07. Here, we note that the estimated volume fraction of copolymer from the ellipsometry data only is 0.029. This is also consistent with a 90–93% hydration of the PNIPAAM layer observed below from the NR analysis discussed below. It is also in broad agreement with a previous QCM-D study on a similar PNIPAAM-*b*-PAMPTMA(+) block copolymer, where a water content of 87% in the polymer layer was found [27]. The consistency in our experimental data also validates the use of Eq. 3 for calculation of ∆*m*. The increase in dissipation is only minor, ∆*D* = 1 × 10^−6^, and it does not seem to vary with the overtone (Fig. 2).

The temperature was thereafter increased to 40 °C, which caused an expected increase in ∆*f*, but only a slight decrease in ∆*D* associated with the collapse of the copolymer layer. Recently, QCM-D has been used to investigate the thermodynamic behavior of surface-bound PNPAAM-based films [49]. These studies showed significantly larger frequency shift and also changes in ∆*D*, which depended on the overtone. Such changes were not observed in the present study. This is probably due to the fact that in the study by Alf et al. [49], the thermoresponsive layer had a higher density and was thicker since the films were prepared via initiated chemical vapor deposition (iCVD) resulting in significantly larger film thickness with mesh sizes of about 60 Å.

After decreasing the temperature to 20 °C, the values returned to those measured at the same temperature previously, verifying the reversibility of the collapse and swelling processes observed above with ellipsometry. At 20 °C, the silica surface with the hydrated copolymer layer was rinsed and then exposed to 0.1 wt% γ-CD solution (Fig. 2). The slight change in ∆*f* to less negative values suggests a loss of wet mass relative the layer without γ-CD at the same temperature (Table 2). This can be interpreted as a loss of water and/or loss of copolymer and supports our observations made using ellipsometry above. The simultaneous decrease in ∆*D* to about zero (i.e., an elastic film), however suggests a stiff (elastic) layer, which can be explained by threading of γ-CD molecules onto the PNIPAAM chains in the creation of the inclusion complexes. The γ-CD molecules are, according to Lazzara et al. [18], threaded in a compact arrangement along the chain with a stoichiometry close to two NIPAAM units per CD molecule determined (using NMR) for bulk solution. Bearing in mind the confinement at an interface, this should be considered to be the maximum amount of CD that can be threaded onto the PNIPAAM chains.

The temperature was then increased to 40 °C, which resulted in higher ∆*f* values compared with those before the addition of γ-CD. The wet mass at the surface therefore decreased to a greater extent than the system in the absence of γ-CD. This result is in consistent with the more dramatic collapse of the polymer layer after its exposure to γ-CD as observed above with ellipsometry. However, only NR has the sensitivity to determine the detailed structure of the interface before and after the interaction.

#### Complex adsorption

**Fig. 3:**
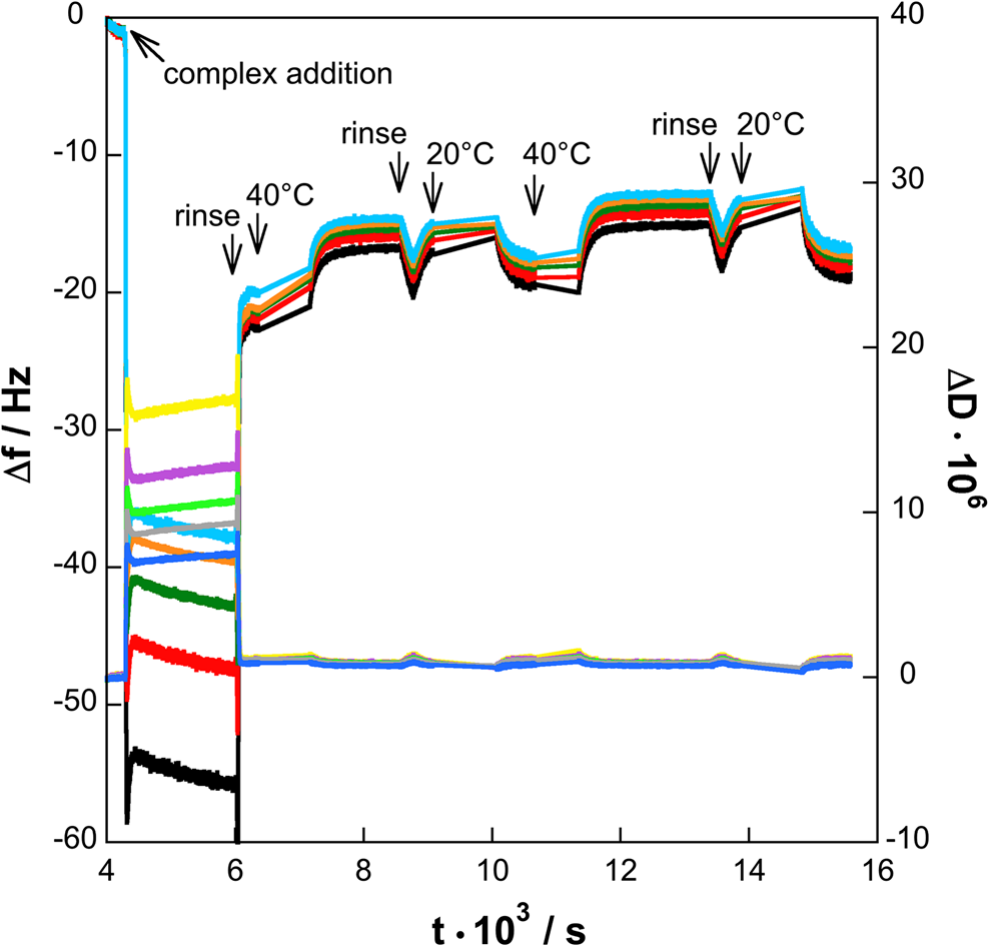
Changes in frequency shifts Δ*f*: from *bottom*, F3 (*black*), F5 (*red*), F7 (*green*), F9 (*turquoise*), F11 (*orange*), and F13 (*light blue*) and dissipation differences Δ*D*: D3 (*yellow*), D5 (*magenta*), D7 (*light green*), D9 (*pink*), D11 (*gray*), and D13 (*blue*) at different overtones (overtone numbers *o*_*n*_ = 3, 5, 7, 9, 11, and 13) of the resonance frequency determined by QCM-D. The γ-CD—PNIPAAM_24_-*b*-PAMPTMA(+)_9_ inclusion complexes were formed in a premixed aqueous solution as described in the text. The times for inclusion complex addition, change in temperature, and rinsing are indicated by *arrows*

A pre-mixed aqueous solution of 5 wt% γ-CD and 0.5 wt% PNIPAAM_24_-*b*-PAMPTMA(+)_9_ containing 1 mM NaCl was exposed to a silica surface to investigate the effects of temperature changes (20 and 40 °C). It has been shown previously that γ-CD can form inclusion complexes with this block copolymer in bulk solution [18]. The reason for using such a high concentration of γ-CD in these experiments was thus to maximize the bulk solution concentration of inclusion complexes and thus limiting the concentration of free copolymer, which is likely to have a higher affinity to the surface as discussed above. Figure 3 presents the changes in ∆*f* and ∆*D* at different resonance harmonics when the silica surface is exposed to inclusion complexes during the temperature cycle 20 → 40 → 20 → 40 → 20 °C as functions of time.

Immediately after the inclusion complexes were exposed to the silica surface, very large changes in ∆*f* and ∆*D* were observed. These striking changes in the different harmonics of the resonance frequency shift, as well as of the dissipation change, are related to a viscoelastic solution effect, which is a result of the high concentration of γ-CD (5 wt%) in the mixed solution compared to 1 mM NaCl solution. The surface was thereafter rinsed with 1 mM NaCl solution (at 6035 s), which led to an increase in ∆*f*. This is mainly due to change of rheological properties of the bulk solution, although some desorption of inclusion complexes cannot be ruled out. The ∆*f* and ∆*D* for the 3rd overtone reach a steady state at 21.2 ± 0.2 Hz and 1.00 ± 0.05 × 10^−6^, respectively (Table 2). The corresponding value of the wet mass is 3.70 ± 0.05 mg m^−2^, which is exactly the same as for the sequential adsorption of complex. If one assumes that the inclusion complexes adsorb from the pre-mixed solution without any dethreading of the γ-CD molecules, the equivalence of the measured value of wet mass in the sequential adsorption experiment provides an indirect indication that in that experiment the γ-CD molecules thread the anchored PNIPAAM chains while anchored at the solid/liquid interface.

The temperature was raised to 40 °C after the rinsing step. The effect of this temperature increase is apparent from Fig. 3 as an increase in ∆*f* to reach a steady-state value of ∆*f* of the 3rd overtone to −16.5 ± 0.1 Hz and ∆*D* = (0.90 ± 0.05) × 10^−6^ and with a corresponding ∆*m* of 2.90 ± 0.05 mg m^−2^ (Table 2). This wet mass at 40 °C is lower than that recorded at 20 °C, which is consistent with the temperature-induced compaction of the PNIPAAM chains being accompanied by a decrease in the hydration.

The temperature was then cycled back to 20 °C then up to 40 °C and finally back to 20 °C. These cycles were accompanied by progressively smaller changes in ∆*f* for the 3rd overtone: to −18.1 ± 0.1 Hz for the first decrease and to 17.4 ± 0.2 Hz for the second decrease with corresponding wet masses of 3.15 ± 0.05 and 3.07 ± 0.05 mg m^2^ (Table 2). On the other hand, we observed no such effects from simply rinsing the interfacial layer at a fixed temperature (constant values of frequency shifts; Fig. 3). These results demonstrate that progressive small losses of material from the interface occur as a result of the temperature cycles. This loss may be attributed to the less stable anchoring of the polymer at the silica surface in the presence of γ-CD, as indicated by the ellipsometry measurements above.

In summary, the QCM-D measurements performed via two different experimental pathways provide indirect evidence of the presence of γ-CD—PNIPAAM_24_-*b*-PAMPTMA(+)_9_ inclusion complexes at the solid/liquid interface and that during the sequential adsorption experiment, the inclusion complexes must have formed in situ at the solid/liquid interface itself. The data also confirm indications from ellipsometry of the lower stability of the mixed system than of the copolymer alone at the interface. These issues are elaborated with complementary information from the NR experiments performed on a longer copolymer below.

### Detailed interfacial structure and composition (NR)

So far, we have reported the changes in adsorbed amount, layer thickness, hydration, and elasticity of copolymer/γ-CD layers at the solid/liquid interface. Nevertheless, it is clear that determination of the layer density profile would help us to understand better the mechanism behind the temperature responsive nature of the interaction. NR measurements were therefore carried out. A copolymer with a longer cationic block (PNIPAAM_41_-*b*-PAMPTMA(+)_24_) was used in these experiments to increase the binding strength of the electrostatic copolymer/silica interaction. The reason for this change is that our ellipsometry data suggest that polymer desorption occurs when γ-CD is added to the system. A longer cationic anchor block would increase the affinity between the cationic copolymer chains and the oppositely charged silica surface and hence reduce desorption of the copolymer inclusion complex from the surface.

**Fig. 4:**
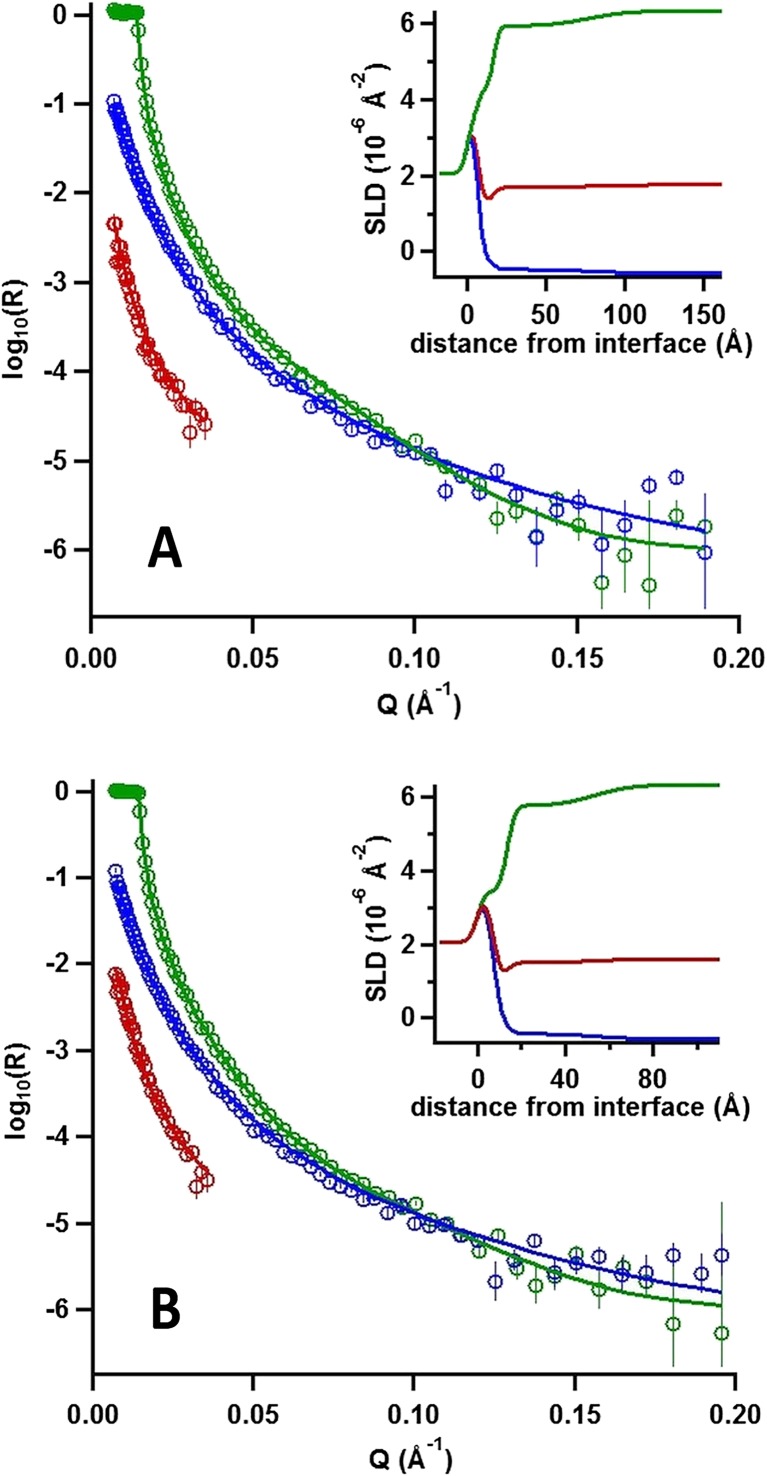
Neutron reflectivity profiles expressed as log(neutron reflectivity, *R*) versus momentum transfer (*Q*) recorded in different solvent contrasts after addition of 0.1 wt% PNIPAAM_41_-*b*-PAMPTMA(+)_24_ copolymer in 1 mM NaCl to silicon substrate at 20 °C (**A**) and 46 °C (**B**). *Symbols*: D_2_O (*green symbols*), H_2_O (*blue symbols*), and a D_2_O/H_2_O mixture (*red symbols*). *Error bars* given as ±std. *Insets*: Variation of the scattering length densities (SLD) as a function of distance from the interface in each case

**Fig. 5:**
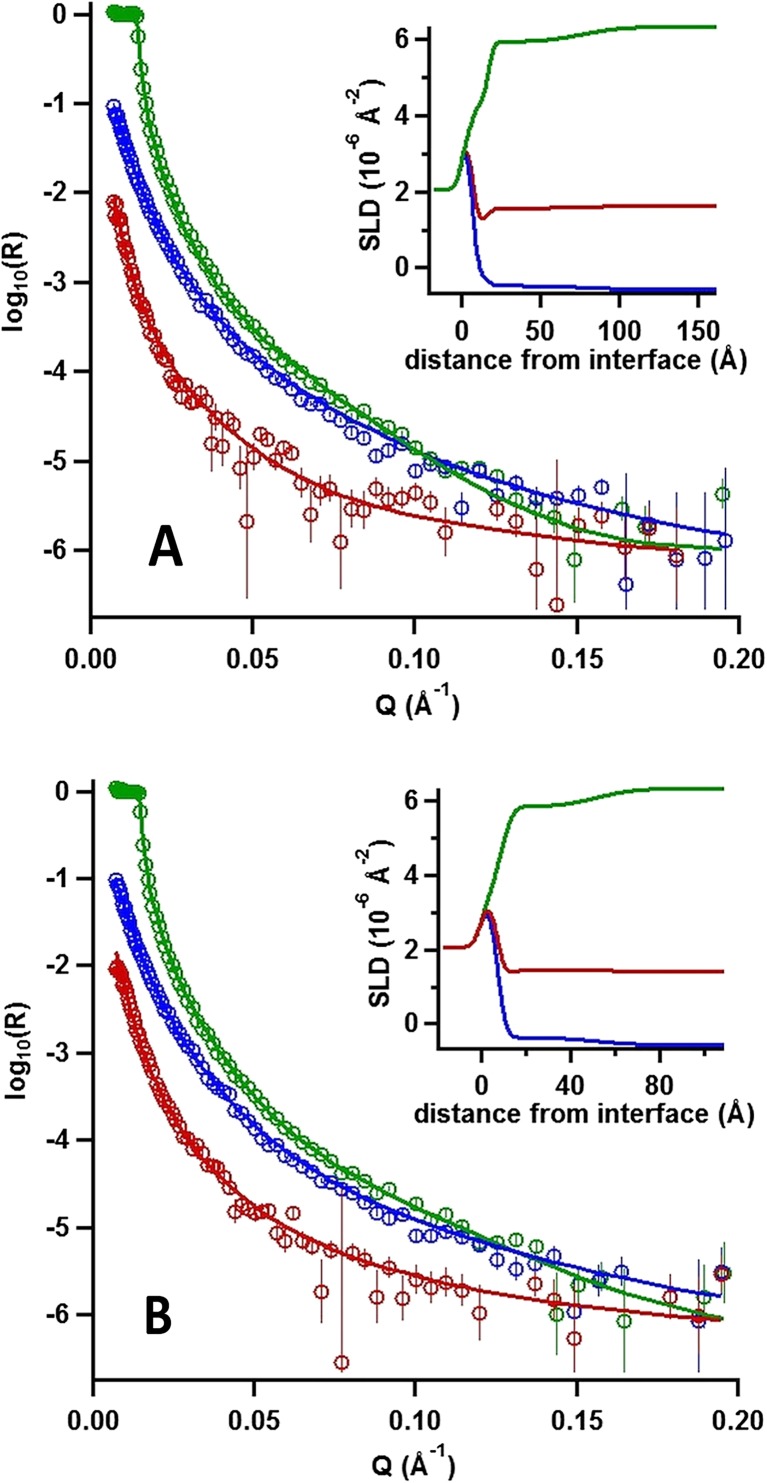
Neutron reflectivity profiles expressed as log(neutron reflectivity, *R*) versus momentum transfer (*Q*) recorded in different solvent contrasts after additions of (i) 0.1 wt% PNIPAAM_41_-*b*-PAMPTMA(+)_24_ copolymer in 1 mM NaCl followed by (ii) 1 wt% γ-CD to silicon substrates at 20 °C (**A**) and after subsequently increasing the temperature to 46 °C (**B**). *Symbols*: D_2_O (*green symbols*), H_2_O (*blue symbols*), and a D_2_O/H_2_O mixture (*red symbols*). *Error bars* given as ±std. *Insets*: Variation of the scattering length densities (SLD) as a function of distance from the interface in each case

As above, first, the adsorption and thermal response of the copolymer, adsorbed from a 0.08 mg mL^−1^ (≈0.1 wt%) PNIPAAM_41_-*b*-PAMPTMA(+)_24_ in 1 mM NaCl aqueous solution, was investigated for which we show the reflectivity profiles, model fits, and resulting scattering length density profiles in Fig. 4. After revealing the effects of adding of 1.0 mg mL^−1^ (≈1.0 wt%) γ-CD (also in 1 mM NaCl aqueous solution), the respective information at the two temperatures is shown in Fig. 5. The solid lines in the figures represent the optimum model fits in three isotopic contrasts to a stratified five-layer model consisting of silicon–silicon oxide cationic anchor PAMTPMA(+) block—extended PNIPAAM block (with or without γ-CD)—aqueous bulk solution. The parameters used in the fit are listed and shown in Table 1, and the parameters obtained from the model fits are listed in Table 3.

**Table 3:** Fitting parameters of five-layer model to neutron reflectivity profiles of macromolecular layers generated by sequential addition of PNIPAAM_41_-*b*-PAMPTMA(+)_24_ copolymer with and without γ-cyclodextrin (γ-CD) measured in D_2_O, H_2_O, and D_2_O/H_2_O mixtures

Parameters	Copolymer 20 °C	Copolymer 46 °C	Copolymer + γ-CD 20 °C	Copolymer + γ-CD 46 °C
SiO_2_				
*d*_ΝR_/Å	7 ± 1	7 ± 1	7 ± 1	7 ± 1
Volume % solvent	20 ± 5	20 ± 5	20 ± 5	20 ± 5
*ξ* (SiO_2_–anchor)/Å	3.0 ± 0.5	3.0 ± 0.5	3.0 ± 0.5	3.0 ± 0.5
Anchor block				
*d*_ΝR_/Å	10 ± 3	6.5 ± 1	10 ± 3	6.5 ± 2
Volume % solvent	65 ± 5	50 ± 5	65 ± 5	75 ± 5
*ξ* (anchor-extended)/Å	3.0 ± 0.5	3.0 ± 0.5	3.0 ± 0.5	3.0 ± 0.5
Extended block				
*d*_NR_/Å	60 ± 6	40 ± 4	60 ± 6	40 ± 4
Volume % water	93 ± 1	90 ± 1	93 ± 1	90 ± 1
*ξ* (extended-solvent)/Å	20 ± 3	13 ± 2	20 ± 3	13 ± 2
γ-CD/PNIPAAM ratio	0 ± 0	0 ± 0	0 ± 0.2	0.5 ± 0.2

*d*_*NR*_ = layer thickness, *ξ* = roughness, *ρ* = scattering length density of macromolecules

The pure PNIPAAM_41_-*b*-PAMPTMA(+)_24_ block copolymer system results in no loss of adsorbed polymer when changing the temperature from 20 and 46 °C. The temperature increase results in a collapse of both the anchor and extended blocks by a factor of one third, and the overall layer thickness (i.e., the sum of the two model layers) is compressed from 70 to 46.5 Å. This observation is qualitatively consistent with the results from ellipsometry and QCM-D on the shorter copolymer described above.

The effects on the experimental NR data of the interaction of γ-CD with the PNIPAAM_41_-*b*-PAMPTMA(+)_24_ copolymer layer are rather small, probably due to the very low coverage of the polymer layer (only 7–10%). As such, the measurements are not very sensitive to small amounts of CD at the interface, and unfortunately, it was not possible to draw firm any conclusions about the amount or location of CD at the interface. Indeed, the data recorded at 20 °C with the lower density of (swollen) polymer result in the same model fit as those without γ-CD to within the experimental errors. Even so, the measurements are very sensitive to the amount and detailed layer structure of the polymer, and the lack of change in the data at 20 °C confirms that effectively the polymer remained strongly bound to the silica after its interaction with γ-CD. This situation is contrary to the results from QCM-D and ellipsometry involving the shorter copolymer, and it follows that the situation is a result of its interaction with γ-CD, possibly due to the stronger anchoring of the copolymer at the surface as the copolymer used in this case had a longer cationic block.

In contrast, at 46 °C, there is loss of half of the PNIPAAM_41_-*b*-PAMPTMA(+)_24_ chains from the surface, presumably an effect of the detachment of the inclusion complexes formed at low temperature. This observation is qualitatively consistent with the data from ellipsometry and QCM-D presented above. It may be that the driving force of the removal of copolymer chains at 46 °C is the hydrophobic interaction between the dehydrated PNIPAAM blocks that come into play once the CD molecules dethread them. We may infer that this interaction overcomes the electrostatic interaction with the surface and desorption takes place. Further, the optimal model fit to the experimental data at 46 °C showed incorporation of a commensurate amount of γ-CD into the PNIPAAM layer. At these very low layer coverages, unfortunately, the resulting changes in the experimental data are rather small and it is not possible to derive the interfacial composition precisely. Nevertheless, additional indirect evidence is provided of the formation of γ-CD—PNIPAAM_41_-*b*-PAMPTMA(+)_24_ inclusion complexes anchored at the solid/liquid interface.

## Conclusions

In the present study, we have investigated the adsorption of thermoresponsive cationic PNIPAAM_*n*_-*b*-PAMPTMA(+)_*m*_ diblock copolymers at the silica/water interface and their resulting interactions with γ-CD molecules. A combination of in situ data from null ellipsometry, QCM-D, and NR provided a thorough characterization of the adsorbed amounts, the layer thickness, hydration and viscoelastic properties, as well as the interfacial structure and composition of the adsorbed copolymer layer in the absence and presence of γ-CD molecules at temperatures below and above the LCST of PNIPAAM. The polymers studied were a relatively short molecule with *m* = 24 and *n* = 9 for ellipsometry and QCM-D, but for the NR experiment involving precious beam time, a longer molecule with *m* = 41 and *n* = 24 was used to enhance the binding strength of the cationic anchor to the hydrophilic silica.

The copolymers adsorb to hydrophilic silica surfaces at 20 °C with a mean layer thickness of 84 Å (ellipsometry) in a conformation that is >90% hydrated (ellipsometry and QCM-D). A near-surface layer of the anchor has a coverage of around one third while an extended layer of the PNIPAAM is five times more hydrated (NR). During rinsing, the layer thickness and adsorbed amount are unchanged. The expected reversible collapse/swelling behavior of the PNIPAAM chains upon temperature changes around the LCST was confirmed.

When γ-CD is exposed to pre-adsorbed copolymer layers at 20 °C, the layer thickness obtained from ellipsometry increases significantly, which is consistent with the specific interaction of γ-CD with the anchored polymer molecules and may also indicate that the anchor block is no longer bound to the silica in such a flat conformation. In our previous work, we found proof of inclusion complex formation between a PNIPAAM diblock copolymer and γ-CD in bulk solution [18]. If γ-CD molecules thread the PNIPAAM chains protruding from the surface as a pearl necklace, they will be forced to stretch to their full length. This physical picture also supports the increase in the layer thickness upon incorporation of γ-CD molecules. There was an accompanied loss of polymer as a result of the interaction, which may be explained in terms of surface-invoked lateral steric repulsion of the formed inclusion complexes. Note that in the case of the NR experiment, when a polymer with a larger cationic anchor was used, no polymer loss was observed simply from the exposure of the film to γ-CD at 20 °C. In this case, it was the heating step to above the LCST that resulted in the loss of half of the polymer from the surface, which can be attributed to the stronger binding to the surface of the longer copolymer.

Temperature plays an important role in this system. The well-known thermoresponsive behavior of PNIPAAM was also observed for the adsorbed copolymer, where protruding PNIPAAM block was found to collapse of the adsorbed polymer layer upon approach of the LCST. What we learned in the present study is that both the PAMPTMA(+) anchor layer and the PNIPAAM extended layer compressed by a factor of about one third (NR). With such a low volume fraction of the PNIPAAM layer, NR was at the limit of its sensitivity to quantify the amount of γ-CD at the interface. Indeed, at 20 °C, the inclusion of γ-CD in the model did not result in an improvement in the fit to the experimental data. Nevertheless, when there was a higher layer density after the film collapsed above the LCST, the model fit improved slightly through the inclusion of γ-CD in the extended PNIPAAM layer, which provided an indication of the formation of inclusion complexes at the solid/liquid interface. Further, indirect evidence came from a comparison of two experiments performed with different pathways using QCM-D, as the interfacial properties resulting from the direct adsorption of inclusion complexes from the bulk and that formed by sequential adsorption of polymer then γ-CD were equivalent.
